# Orthodontic clinicians’ attitudes and knowledge of dentogingival aesthetics: A cross-sectional survey of BOS members

**DOI:** 10.1177/14653125211034878

**Published:** 2021-08-11

**Authors:** Eman Ajrash, Andrew T DiBiase, Nikolaos Pandis, Martyn T Cobourne, Jadbinder Seehra

**Affiliations:** 1 Department of Orthodontics, Faculty of Dentistry, Oral and Craniofacial Sciences, King’s College London, London, UK; 2 Department of Orthodontics, Maxillofacial Unit, William Harvey Hospital, Willesborough, Ashford, UK; 3 Department of Orthodontics and Dentofacial Orthopedics, Dental School/Medical Faculty, University of Bern, Bern, Switzerland

**Keywords:** periodontic interface, biomechanics, adult orthodontics

## Abstract

**Objective:**

To assess orthodontic clinicians’ knowledge and attitudes towards dentogingival aesthetics and to explore characteristics that predict the knowledge of dentogingival aesthetics.

**Design:**

Cross-sectional questionnaire

**Setting:**

On-line survey of members of the British Orthdontic Society.

**Materials and Methods:**

An 11-item online questionnaire was sent to orthodontic practitioners for completion. The questionnaire covered respondent demographics and questions relating to both knowledge and attitudes towards dentogingival aesthetics (six parameters). Descriptive statistics were calculated for study characteristics and summary values for the survey items. Responses to the eight knowledge-based questions were converted to a binary outcome (correct and incorrect answer). The maximum score that could be achieved was eight. Multivariable modelling was used in order to examine associations between the study characteristics and the aggregate score.

**Results:**

A total of 252 responses were obtained resulting in a response rate of 17%. Within this cohort, the respondents were primarily women (52.8%) and aged 30–40 years (35.7%). The mean score for the eight knowledge-based questions was 3.8 ± 1.8 (range = 0−8). Knowledge of the ideal gingival margin position of the anterior teeth was high (92.4%). Knowledge of the other five dentogingival aesthetic parameters was variable. In the multivariable analysis, lower knowledge scores were predicated by respondents who did not have a special interest in dental aesthetics (−0.54; 95% confidence interval [CI] = −1.01 to −0.07; *P* = 0.02), who could not recall attending courses, lectures or seminars on dental aesthetics in the past five years (−0.80; 95% CI = −1.43 to −0.17; *P* = 0.01) and with increasing age (−0.43; 95% CI = −0.62 to −0.23; *P* < 0.001).

**Conclusion:**

Knowledge of ideal dentogingival parameters is generally suboptimal among orthodontists in the UK. The reported lack of knowledge of the ideal dentogingival parameters may also influence respondents’ attitudes towards the importance of dentogingival aesthetics. Further teaching or courses related to dentogingival aesthetics is desired by orthodontic clinicians.

## Introduction

**Figure 1.**
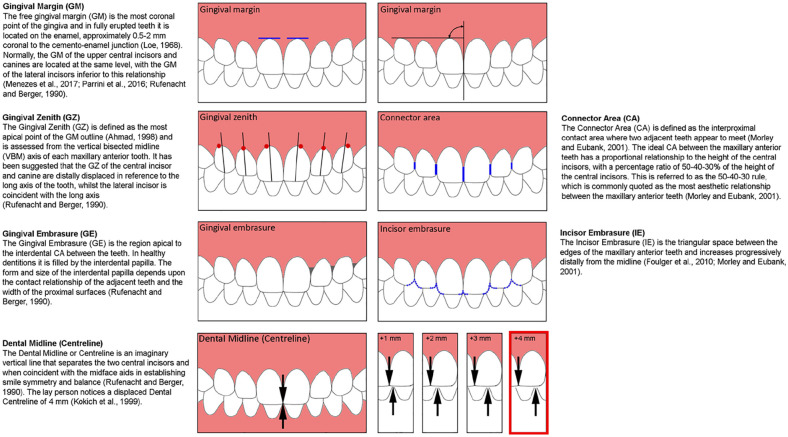
Dentogingival parameters.

An aesthetic smile is significantly influenced by the quality of the component dental and gingival elements and their conformity to accepted norms (Rufenacht and Berger, 1990). Healthy gingival tissues tend to have specific morphological traits, which include knife-edged gingival margins tightly adapted to the teeth with a keratinised, stippled or smooth pink surface (Rufenacht and Berger, 1990). Based on clinical studies of patient samples, a number of collective dentogingival (DG) characteristics have also been recognised as key determinants in achieving an aesthetic smile (Figure 1).

The orthodontist usually aims to achieve an ideal occlusion, but this can differ from the patients’ aim, which is often driven by aesthetic improvements, particularly in adults (Gochman, 1975; Shaw, 1981; Tulloch et al., 1984). In addition, orthodontic treatment can influence DG architecture resulting in non-ideal gingival aberrations. Alignment of maxillary incisors with triangular crown morphology and some loss of attachment (Atherton, 1970), labial movement of teeth during the management of Class II division 2 malocclusion, palatally impacted canines or mesially rotated teeth (Sharma and Park, 2010) can all lead to open gingival embrasures as a result of apical movement of the gingival tissues (Kandasamy et al., 2007). An open gingival embrasure or ‘dark triangle’ between the maxillary central incisors has been reported to be present in 38% and 42% of adult and adolescent patients, respectively, after orthodontic treatment (Burke et al., 1994; Kurth and Kokich, 2001).

Due to the great emphasis being placed on smile aesthetics over the last two decades, it is crucial to ensure that all aspects of a smile are considered—including an assessment of ideal DG positions. The importance of DG features in determining facial and smile aesthetics have been highlighted (Malheiros et al., 2018). Within the literature, an assessment of orthodontic clinicians’ knowledge of ideal DG parameters is lacking. Therefore, the primary aim of this study was to assess the knowledge and attitudes of orthodontic clinicians towards DG aesthetics. In addition, characteristics that predict the knowledge of DG aesthetics were explored.

## Materials and Methods

The study was a cross-sectional online survey distributed to members of the British Orthodontic Society (BOS). Ethical approval was obtained from King’s College London College Research Ethics Committee (MRSU-19/20-20075) and permission from the BOS Clinical Governance directorate before distribution of the survey. A checklist for good practice in the conduct of surveys was used to allow for high standards of research and results of credible value (Kelley et al., 2003). In addition, this study is reported in accordance with the Checklist for Reporting Results of Internet E-Surveys (CHERRIES) (Eysenbach, 2004) (Supplemental Table 1).

### Participants and mode of collection

Orthodontic members of the following membership groups of the BOS were invited to participate: Consultant Orthodontic Group (COG); Community Group (CG); Orthodontic Specialists Group (OSG); University Teachers Group (UTG); Practitioner Group (PG); and Trainee Grades Group (TGG). This sample represents orthodontists with a range of orthodontic experience and background. An invitation email was sent with a link to the survey and a participant information sheet outlining the purpose of the survey, data management and participant commitment. Consent was indicated by participation in the survey with the ability to withdraw at any time by exiting the survey. Participation in this survey was completely voluntary and incentives were not provided. The survey was sent to 1478 email addresses on the BOS mailing list; however, it was noted that some clinicians are members of more than one BOS group and some email addresses would not receive the email. Therefore, the percentage response provided in the results is only an estimate. The initial email was followed by two reminder emails at three-week and nine-week intervals. The survey was open for 12 weeks from July until October 2020 and was administered and collected using Jisc Online Surveys (onlinesurveys.ac.uk).

### Development and validation of the questionnaire

The process for developing the survey involved the following: (1) setting a clear research question and determining the research objective based on an extensive literature search on DG features of the anterior dentition that can be influenced by orthodontic treatment, that could be objectively measured and can have an influence on smile aesthetics, (2) item generation, questionnaire formatting, testing and validation; and (3) content validity (Lynn, 1986; Tsang et al., 2017) was assessed by three consultant orthodontists with a range of clinical experience and who were familiar with the research aims. The final 11 questions were divided into two categories (knowledge and attitudes), and were determined by consensus agreement between the authors (Supplementary Table 2). The knowledge questions were focused on the recognised key determinants in achieving an aesthetic smile (Sarver, 2004). Usability and technical functionality of the electronic questionnaire was tested before distribution of the questionnaire by piloting the survey.

### Data collection

Respondent demographics and survey responses were collected and entered into a password-protected Microsoft Excel® (Microsoft, Redmond, WA, USA) data collection sheet.

### Statistical analysis

Descriptive statistics were calculated for study characteristics and summary values for the survey items. Responses to the eight knowledge-based questions were converted to a binary outcome (correct and incorrect answer). The maximum score that could be achieved was 8. The aggregate score of the eight knowledge-based items was the dependent variable in the series of univariable linear regression models in which we examined potential associations between the study characteristics and the aggregate score. The significant variables were added into a multivariable model. A two-tailed *P* value of 0.05 was considered statistically significant. Statistical analyses were performed using STATA software version 16.1 (Stata Corporation, College Station, TX, USA) and R Software version 3.6.1 (R Foundation for Statistical Computing, Vienna, Austria).

## Results

A total of 252 responses were received from 1478 emails, giving a response rate of 17%. Of the respondents, 52.8% were women and 47.2% were men. The sample consisted mainly of individuals aged 30−40-years (35.7%). Most respondents had completed their dental qualification (75.4%) and orthodontic specialty training (89.1%) in the UK or Ireland with an awarded Master’s degree (research and taught combined, 74.3%). When respondents were asked if they had any special interest in dental aesthetics, 42.5% of the sample disclosed that they did and 45.6% had attended courses, lectures or seminars on dental aesthetics in the past five years (Table 1).

**Table 1.** Participant characteristics (n = 252).

Characteristics	n (%)
Sex	
Male	117 (47.2)
Female	131 (52.8)
Age (years)	
<30	14 (5.6)
30–40	90 (35.7)
41–50	64 (25.4)
51–60	65 (25.4)
>60	19 (7.5)
Country of dental qualification	
UK and Ireland	190 (75.4)
EU	10 (4.0)
Other	52 (20.6)
Country of orthodontic qualification	
UK and Ireland	220 (89.1)
EU	9 (3.6)
Other	18 (7.3)
Level of orthodontic qualification	
Certificate	13 (5.2)
Diploma	15 (6.0)
Masters (taught)	125 (50.2)
Masters (research)	60 (24.1)
Doctorate (taught)	14 (5.6)
Doctorate (research)	22 (8.8)
Special interest in dental aesthetics	
Yes	107 (42.5)
No	145 (57.5)
Courses in past five years	
Yes	115 (45.6)
No	98 (38.9)
I don’t recall	39 (15.5)

### Knowledge of DG aesthetics

Knowledge of the ideal gingival margin (GM) position of the anterior teeth was high (92.4%). Most participants were also aware of the ideal incisor embrasure (IE) relationship of the anterior teeth (61.5%). Knowledge of the ideal gingival embrasure (GE) and ideal connector area (CA) was < 50%. Approximately one-third of the sample were aware of the accepted limit for dental midline deviation for laypeople (31.4%). A slightly greater proportion of participants (34.6%) believed that the dental midline can be deviated by only 2 mm before it is detected by the lay person. Awareness of gingival zenith (GZ) position was also deficient, with 41.6% correctly identifying the ideal GZ position of the central incisor and only 27.4% correctly identifying the GZ position of the maxillary lateral incisor and canine. A large proportion of respondents acknowledged that they did not know the answers to some of the questions. The parameter that participants were least familiar with was the CA, with 41.5% stating that they do not know the answer (Table 2).

**Table 2.** Responses to knowledge-based questions (correct responses highlighted in red) (n = 252).

Question	Response options						Supporting reference for correct answer
What is the ideal GZ position relative to the vertical bisected midline for each of the following teeth?							
Maxillary central incisor	Mesial 11 (4.7)	Coincident 74 (29.2)	Distal 105 (41.6)	I don’t know 62 (24.5)			Rufenacht and Berger, 1990
Maxillary lateral incisor	Mesial 24 (9.5)	Coincident 68 (26.9)	Distal 91 (36.2)	I don’t know 69 (27.4)			Rufenacht and Berger, 1990
Maxillary canine	Mesial 23 (9.1)	Coincident 88 (34.9)	Distal 69 (27.4)	I don’t know 72 (28.6)			Rufenacht and Berger, 1990
Which of the statements is true for the ideal GM level of the maxillary incisor teeth	Central, lateral and canine should all be at the same level 2 (0.8)	Central and lateral incisors should be at the same level and the margin of the canine should be lower 1 (0.4)	Central and canine should be at the same level and the margin of the lateral incisor should be at a lower level 233 (92.5)	Central should be higher than the margin of the lateral incisor and canine 9 (3.7)	I don’t know 7 (2.7)		Rufenacht and Berger, 1990
An open GE space (black triangle) is noticeably less aesthetic if it is more than	1 mm 65 (25.8)	2 mm 120 (47.6)	3 mm 32 (12.8)	+4 mm 10 (3.9)	I don’t know 25 (9.9)		Kokich et al., 1999
What is the ideal CA ratio between the maxillary central incisor, lateral incisor and canine?	50:40:30 123 (48.9)	40:30:20 3 (1.3)	30:40:50 18 (7.2)	50:50:50 4 (1.6)	I don’t know 104 (41.5)		Morley and Eubank, 2001
Ideally the IE space between the maxillary central incisors, the central incisor and lateral incisors and the lateral incisors and canines should:	Increase progressively distally from the midline 155 (61.5)	Decrease progressively distally from the midline 22 (8.7)	Decrease progressively mesially from the midline 2 (0.8)	Be equal in size 11 (4.4)	I don’t know 62 (24.6)		Morley and Eubank, 2001
The dental centreline can be deviated by ______ before it is noticed by laypeople?	1 mm 12 (4.7)	2 mm 87 (34.6)	3 mm 45 (17.8)	4 mm 79 (31.4)	+5 mm 11 (4.4)	I don’t know 18 (7.1)	Kokich et al., 1999

Values are given as n (%).

CA, connector area; GE, gingival embrasure; GM, gingival margin; GZ, gingival zenith; IE, incisor embrasure.

**Figure 2.**
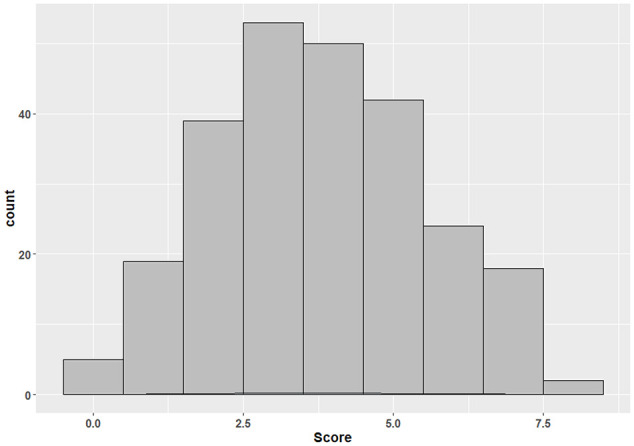
Distribution of aggregate knowledge scores (n = 252).

**Figure 3.**
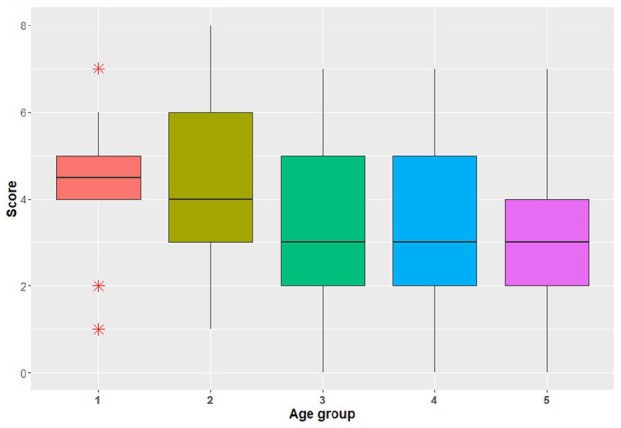
Distribution of aggregate knowledge scores per age group (1 = <30 years, 2 = 30–40 years, 3 = 41−50 years, 4 = 51−60 years and 5 = >60 years).

**Figure 4.**
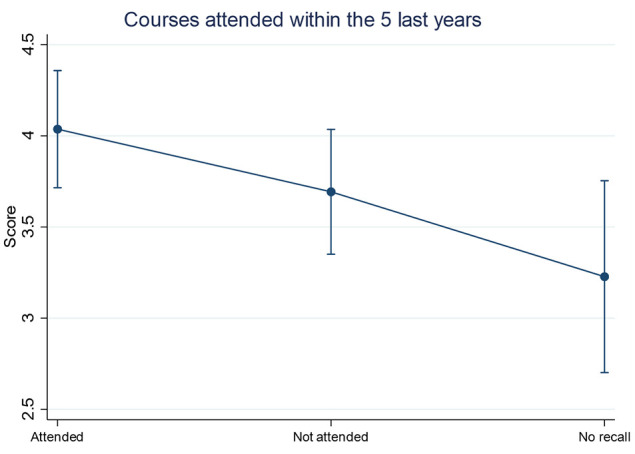
Predicted aggregate mean knowledge scores for participants who attended any courses, lectures or seminars on dental aesthetics in the past five years from the multivariable analysis.

The mean score for the eight knowledge-based questions was 3.8 ± 1.8 (range = 0–8) (Figure 2). Lower knowledge scores were obtained in participants with increasing age while those aged 30–40 years had the highest mean scores (Figure 3). In the multivariable analysis, lower knowledge scores were predicated by respondents who did not have a special interest in dental aesthetics (–0.54; 95% confidence interval [CI] = –1.01 to −0.07; *P* = 0.02), who could not recall attending courses, lectures or seminars on dental aesthetics in the past five years (−0.80; 95% CI = −1.43 to −0.17; *P* = 0.01) and with increasing age (−0.43; 95% CI = −0.62 to −0.23; *P* < 0.001) (Table 3). Participants who had attended courses, lectures or seminars on dental aesthetics in the past five years also had higher knowledge scores compared to those who either had not or who did not recall after adjusting for age and special interest in aesthetics (Figure 4).

**Table 3.** Linear regression and 95% CIs for the effect of gender, level of orthodontic qualification, special interest in dental aesthetics, courses in past five years and age on aggregate knowledge score.

Predictor variables	Category	Univariable analysis		Multivariable analysis	
		Coef. (95% CI)	*P* value		*P* value
Sex	Male	Baseline (reference)			
	Female	0.10 (–0.33 to 0.55)	0.64		
Level of orthodontic qualification	Certificate	Baseline (reference)			
	Diploma	–0.24 (−1.53 to 1.05)	0.72		
	Masters (taught)	0.34 (–0.65 to 1.34)	0.49		
	Masters (research)	0.85 (–0.18 to 1.90)	0.11		
	Doctorate (taught)	1.12 (–0.19 to 2.44)	0.10		
	Doctorate (research)	0.46 (–0.73 to 1.66)	0.45		
Special interest in dental aesthetics	Yes	Baseline (reference)			
	No	–0.90 (−1.33 to −0.47)	<0.001	–0.54 (−1.01 to −0.07)	0.02
Courses in past five years	Yes	Baseline (reference)		Reference	
	No	–0.65 (−1.12 to −0.18)	<0.01	–0.34 (−0.83 to 0.14)	0.17
	I don’t recall	–0.91 (−1.54 to −0.18)	<0.01	–0.80 (−1.43 to −0.17)	0.01
Age	Per unit	–0.46 (−0.65 to −0.26)	<0.001	–0.43 (−0.62 to −0.23)	<0.001

CI, confidence interval.

### Attitudes towards DG aesthetics

When asked about the importance of occlusion and smile aesthetics when assessing orthodontic treatment outcomes, most orthodontists believed that these were very important: 75.0% and 78.6%, respectively. In contrast, only 38.1% considered DG aesthetics to be of importance. An approximately equal number of respondents believed that the DG parameters mentioned in the survey were very important (46.4%) or fairly important (47.2%) on overall smile aesthetics. Half of the responders believed that orthodontic treatment can moderately influence DG aesthetics, while only 37.3% believed orthodontic treatment can greatly influence DG aesthetics (Table 4).

**Table 4.** Responses to attitude-based questions (n = 252).

Attitude questions	Responses				
When assessing orthodontic treatment outcomes, in your opinion how important is it the consider the following features:	Very important	Fairly important	Slightly important	Not important	
Occlusion	189 (75.0)	61 (24.2)	2 (0.8)	-	
Smile aesthetics	198 (78.6)	52 (20.6)	1 (0.4)	1 (0.4)	
Dentogingival aesthetics	96 (38.1)	144 (57.1)	11 (4.4)	1 (0.4)	
In your opinion how important are the above mentioned dentogingival features on overall smile aesthetics?	117 (46.4)	119 (47.2)	16 (6.4)	-	

In your opinion, how much can orthodontic treatment influence dentogingival aesthetics?	Greatly	Moderately	Slightly	No effect	
	94 (37.3)	126 (50.0)	32 (12.7)	-	

Values are given as n (%).

**Figure 5.**
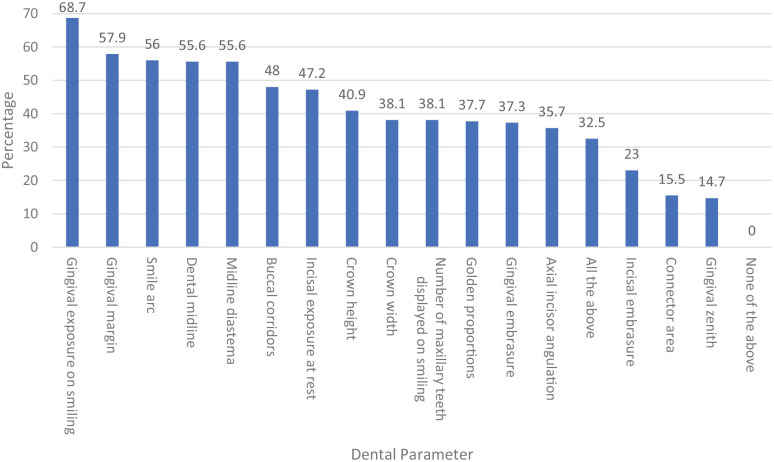
Dental parameters in order of highest to lowest impact on dental aesthetics as perceived by orthodontists.

When asked about the features that can have the greatest aesthetic impact on dental aesthetics, gingival exposure on smiling was highest ranked by 68.7% of respondents. This was followed by GM (57.9%), smile arc (56.0%), dental midline (55.6%), midline diastema (55.6%), buccal corridors (48.0%), incisal exposure at rest (47.2%), crown height (41.0%), crown width (38.1%), number of maxillary teeth displayed on smiling (38.1%), golden proportions (37.7%), GE (37.3%) and axial incisor angulation (35.7%). The parameters that were rated of lowest impact on dental aesthetics were IE (23%), CA (15.5%) and GZ (14.7%). However, 32% of participants specified that all the included parameters have a remarkable impact on dental aesthetic (Figure 5).

A total of 60 respondents took the opportunity to leave additional comments, which provided useful insight on participants opinions and thoughts. Comments were varied, with some believing DG aesthetics not to be of great importance as they are often not visible, while others suggested that the relative importance of these features will depend on patient preferences and concerns. The most common comments were those expressing lack of knowledge on DG aesthetics and the need for further emphasis to be placed as part of orthodontic training. Many orthodontists expressed enthusiasm to learn more on dental aesthetics (Table 5).

**Table 5.** Free-text comments by respondents.

Theme	Comments
Lack of knowledge	• Never heard of most of it. • I’m not sure of the definition of these terms! • Some of the terminology unfamiliar to me. • Sad I am so ignorant with some of the information!! • I am not familiar with some of the terminology used in this questionnaire so was not confident I was interpreting the questions correctly. • I have not had any training in this area and do not have much knowledge of the subject.
Interest and training on DG aesthetics	• Please give us the results of this study when you get them! • I now feel under-informed re anterior aesthetics, some CPD needed! • I would be very interested in further education on smile design for orthodontists – many of the courses that cover this are in restorative courses, • There could be more emphasis and teaching on DG aesthetics in current specialty training. I feel that we get taught the basics of the gingival heights and smile arc and midlines but there is much less teaching on the GZ and connector area.
Difference between laypeople and orthodontists	• Big difference between ortho clinicians and lay people in recognising mild to moderate variation from ideal. • My view on DG aesthetics can be radically different to a patient’s.
Patient factors	• The relative importance on many of these features will depend on the smile line. • Some of the importance related to GMs and GZ will be dependent on the extent to which these are visible when smiling.
Not perceived as priority	• A lot of this cosmetic smile design stuff is for those with time on their hands to address minutiae. • While the above features have a significant impact on dental aesthetics, my role as an NHS employee is to address health issues and dwelling on aesthetics would likely move my specialty out of healthcare.

CPD, continuing professional development; DG, dentogingival; GM, gingival margin; GZ, gingival zenith.

## Discussion

The results of this survey reveal that other than knowledge of GM position associated with the maxillary anterior teeth, DG aesthetic knowledge was generally suboptimal among orthodontists. The parameters that were least-rated as having an impact on dental aesthetics were IE, CA and GZ. This potentially reflects lack of knowledge on the ideal characteristics of these features, and it can be postulated that these parameters may have not been rated highly because most respondents were unfamiliar with them. This can also be confirmed by the fact that just under half of the participants stated that they do not know the ideal CA ratio, and almost one-quarter did not know the ideal IE pattern and GZ position of the anterior teeth.

However, respondents believed that the DG features mentioned in the survey were very important or fairly important on overall smile aesthetics. This signifies that orthodontists acknowledge the importance of these features yet often fail to recognise their ideal characteristics. This could be due to lack of training on DG aesthetics as indicated in the free-text comments. Many respondents expressed that they felt that orthodontic training should have more emphasis on dental aesthetics and conveyed their interest in further education on smile aesthetics. Our results show that further education could be of benefit in improving knowledge on DG aesthetics as respondents who had attended courses, lectures and seminars on dental aesthetics in the past five years obtained higher knowledge scores. Indeed, it has been reported that engaging in continuing professional development is effective and successful in resulting in new knowledge or skills and allows development of a wider knowledge base than that which can be provided by specialty training alone (Barnes et al., 2012). It may also be reflected by the fact that the orthodontist has less direct influence on gingival aesthetics in contrast to tooth position. On this basis, it should be recognised that the ideal DG aesthetics may not be achievable using orthodontic mechanics alone, and input from other dental specialists may be required. Similarly, a better knowledge of these orthodontic limitations will help the orthodontist (and patient) as part of the consent process.

There is large variation in the literature on what an acceptable centreline deviation is, with most studies quoting an accepted limit in the range of 2–4 mm (Beyer and Lindauer, 1998; Cardash et al., 2003; Johnston et al., 1999; Kokich Jr et al., 1999; Pinho et al., 2007). Similarly, most respondents recorded the GZ position of the lateral incisor to be distal and that of the canine to be coincident to the vertical bisected midline. This mirrors previous findings that the GZ position of the central incisor is approximately 1 mm distal, while that of the lateral incisor is 0.4 mm distal and that of the canine coincident with the vertical bisected midline (Chu et al., 2009). Classically, the GZ of the central incisor and canine are distally displaced in reference to the long axis of the tooth, while that of the lateral incisor is coincident with the long axis. This is based on clinical and study model observations and is the most commonly cited ideal GZ position in the literature (Rufenacht and Berger, 1990).

To our knowledge, there is no previous study that has assessed knowledge of dental aesthetics in the orthodontic population. A survey on knowledge of dental aesthetics among general dentists, house surgeons and dental specialists found 92% of the sample to have satisfactory knowledge on anterior dental aesthetics while only half of the sample had satisfactory knowledge on gingival aesthetics. Knowledge of gingival aesthetics was higher among prosthodontists, periodontists and restorative dentists compared to other dental categories, such as orthodontists. It was also found that dentists with > 10 years of clinical experience were more likely to have satisfactory knowledge of gingival aesthetics (Raja et al., 2016). Although our study did not find an association between year of graduation and knowledge, it did reveal lower knowledge scores in respondents with increasing age indicating that increased clinical experience does not necessarily correlate to increased knowledge of DG aesthetics. In fact, all comments specifying that the respondent was unfamiliar with the parameters in question were submitted by clinicians that had obtained their orthodontic specialty training before 2005. Conversely, those aged 30–40 years had the highest knowledge scores. This could mean that undertaking more recent specialty training provides more guidance on DG aesthetics or that younger clinicians are more interested in achieving the most aesthetic results. It is also an indication of the greater importance placed on DG aesthetics in the literature over the last two decades.

The sample was a convenience sample limited to BOS members, which may limit the generalisability of the findings of this survey. Surveys can also be associated with several shortcomings, such as unconscious responses by respondents whereby answers are chosen before fully reading the question or before considering all multiple-choice options as well as answers based on guesses, affecting the reliability of the data. To minimise the chances of participants guessing, an ‘I don’t know’ option was included. This option is suitable when a respondent’s knowledge is being sought to acknowledge respondent uncertainty (Stone,1993). Re-testing a subsample after a period of time to assess if answers remain consistent across repeated administration of the same survey would be unreliable. This is because our research assesses knowledge, and participants may look for or learn the correct answers after administration of the initial survey leading to inconsistent results. The response rate was low at 17% but comparable to the response rates of orthodontists who are members of the BOS using web-based surveys. Based on previous surveys, the average response rate for BOS members is in the range of 14%–19% (Barber et al., 2020; Fleming et al., 2018; Oliver et al., 2020; Sandler et al., 2019). Reasons for low response rates include lack of time, lack of interest or survey fatigue, where participants are flooded with surveys, making them less inclined to participate (Weiner and Dalessio, 2006). This may also occur during the questionnaire if the questionnaire is too long or complicated. Longer surveys have been found to have lower response and completion rates and it is generally recommended to keep the time taken to complete the survey < 5 min (Nakash et al., 2006). We ensured that the average time taken to complete the survey would be < 3 min. Furthermore, as 45.6% of survey participants had attended courses in DG aesthetics, there may be an element of bias in the reported results.

## Conclusions

This study has provided valuable insight on both the knowledge and attitude of orthodontists regarding DG aesthetics. Knowledge of ideal DG parameters is generally suboptimal among orthodontists in the UK. Respondents who have a special interest in dental aesthetics and attended courses, lectures and seminars related to dental aesthetics within the last five years achieved higher knowledge scores. The reported lack of knowledge of the ideal DG parameters may also influence respondents’ attitudes towards the importance of DG aesthetics. Further teaching or courses related to DG aesthetics is desired by orthodontic clinicians.

## Supplemental Material

sj-docx-1-joo-10.1177_14653125211034878 – Supplemental material for Orthodontic clinicians’ attitudes and knowledge of dentogingival aesthetics: A cross-sectional survey of BOS members Click here for additional data file. Supplemental material, sj-docx-1-joo-10.1177_14653125211034878 for Orthodontic clinicians’ attitudes and knowledge of dentogingival aesthetics: A cross-sectional survey of BOS members by Eman Ajrash, Andrew T DiBiase, Nikolaos Pandis, Martyn T Cobourne and Jadbinder Seehra in Journal of Orthodontics

## References

[bibr1-14653125211034878] Ahmad I (1998) Geometric considerations in anterior dental aesthetics: restorative principles. Practical Periodontics and Aesthetic Dentistry 10: 813–22. 10093545

[bibr2-14653125211034878] Atherton JD (1970) The gingival response to orthodontic tooth movement. American Journal of Orthodontics 58: 179–186. 5269763 10.1016/0002-9416(70)90071-0

[bibr3-14653125211034878] Barber SK Ryan F Cunningham SJ (2020) Knowledge of, and attitudes to, shared decision-making in orthodontics in the UK. Journal of Orthodontics 47: 294–302. 32693666 10.1177/1465312520941526

[bibr4-14653125211034878] Barnes E Bullock AD Bailey SE Cowpe JG Karaharju-Suvanto T (2012) A review of continuing professional development for dentists in Europe. European Journal of Dental Education 16: 166–178. 22783843 10.1111/j.1600-0579.2012.00737.x

[bibr5-14653125211034878] Beyer JW Lindauer SJ (1998) Evaluation of dental midline position. Seminars in Orthodontics 4: 146–152. 10.1016/s1073-8746(98)80016-9 9807151

[bibr6-14653125211034878] Burke S Burch JG Tetz JA (1994) Incidence and size of pretreatment overlap and posttreatment gingival embrasure space between maxillary central incisors. American Journal of Orthodontics and Dentofacial Orthopedics 105: 506–511. 8166102 10.1016/S0889-5406(94)70013-3

[bibr7-14653125211034878] Cardash HS Ormanier Z Laufer B-Z (2003) Observable deviation of the facial and anterior tooth midlines. The Journal of Prosthetic Dentistry 89: 282–285. 12644804 10.1067/mpr.2003.68

[bibr8-14653125211034878] Chu SJ Tan JH Stappert CF Tarnow DP (2009) Gingival zenith positions and levels of the maxillary anterior dentition. Journal of Esthetic and Restorative Dentistry 21: 113–120. 19368601 10.1111/j.1708-8240.2009.00242.x

[bibr9-14653125211034878] Eysenbach G (2004) Improving the quality of Web surveys: the Checklist for Reporting Results of Internet E-Surveys (CHERRIES). Journal of Medical Internet Research 6: e34. 15471760 10.2196/jmir.6.3.e34 PMC1550605

[bibr10-14653125211034878] Fleming PS Cunningham SJ Benson PE Jauhar P Millett D (2018) Extraction of premolars for orthodontic reasons on the decline? A cross-sectional survey of BOS members. Journal of Orthodontics. DOI: 10.1080/14653125.2018.1517470. 30192715

[bibr11-14653125211034878] Foulger TE Tredwin CJ Gill DS (2010) The influence of varying maxillary incisal edge embrasure space and interproximal contact area dimensions on perceived smile aesthetics. British Dental Journal 209: E4. 20706227 10.1038/sj.bdj.2010.719

[bibr12-14653125211034878] Gochman DS (1975) The measurement and development of dentally relevant motives. Journal of Public Health Dentistry 35: 160–164. 1057022 10.1111/j.1752-7325.1975.tb00706.x

[bibr13-14653125211034878] Johnston CD Burden DJ Stevenson MR (1999) The influence of dental to facial midline discrepancies on dental attractiveness ratings. European Journal of Orthodontics 21: 517–522. 10565092 10.1093/ejo/21.5.517

[bibr14-14653125211034878] Kandasamy S Goonewardene M Tennant M (2007) Changes in interdental papillae heights following alignment of anterior teeth. Australian Orthodontic Journal 23: 16–23. 17679530

[bibr15-14653125211034878] Kelley K Clark B Brown V Sitzia J (2003) Good practice in the conduct and reporting of survey research. International Journal for Quality in Health Care 15: 261–266. 12803354 10.1093/intqhc/mzg031

[bibr16-14653125211034878] Kokich VO Jr Asuman Kiyak H Shapiro PA (1999) Comparing the perception of dentists and lay people to altered dental esthetics. Journal of Esthetic and Restorative Dentistry 11: 311–324. 10.1111/j.1708-8240.1999.tb00414.x 10825866

[bibr17-14653125211034878] Kurth JR Kokich VG (2001) Open gingival embrasures after orthodontic treatment in adults: prevalence and etiology. American Journal of Orthodontics and Dentofacial Orthopedics 120: 116–123. 11500652 10.1067/mod.2001.114831

[bibr18-14653125211034878] Löe H (1968) Periodontium. In: Goldman HM Cohen HW (eds) Periodontal Therapy. 4th ed. St Louis, MO: Mosby, pp.1.

[bibr19-14653125211034878] Lynn MR (1986) Determination and quantification of content validity. Nursing Research 35: 382–385. 3640358

[bibr20-14653125211034878] Malheiros AS Brito AC Gurgel JA Bandeca MC Borges AH Hayashida TM , et al (2018). Dentogingival alterations and their influence on facial and smile attractiveness. Journal of Contemporary Dental Practice 19: 1322–1328. 30602635

[bibr21-14653125211034878] Menezes EBC Bittencourt MAV Machado AW (2017) Do different vertical positions of maxillary central incisors influence smile esthetics perception? Dental Press Journal of Orthodontics 22: 95–105. 28658361 10.1590/2177-6709.22.2.095-105.oar PMC5484275

[bibr22-14653125211034878] Morley J Eubank J (2001) Macroesthetic elements of smile design. Journal of the American Dental Aasociation 132: 39–45. 10.14219/jada.archive.2001.0023 11194397

[bibr23-14653125211034878] Nakash RA Hutton JL Jørstad-Stein EC Gates S Lamb SE (2006) Maximising response to postal questionnaires–a systematic review of randomised trials in health research. BMC Medical Research Methodology 6: 5. 16504090 10.1186/1471-2288-6-5 PMC1421421

[bibr24-14653125211034878] Oliver GR Lynch CD Fleming PS (2020) What I wish I’d learned as an orthodontic trainee: an online survey of British Orthodontic Society members concerning postgraduate training experiences. Journal of Orthodontics 47: 116–128. 32052682 10.1177/1465312520904367 PMC7498909

[bibr25-14653125211034878] Parrini S Rossini G Castroflorio T Fortini A Deregibus A Debernardi C (2016) Laypeople’s perceptions of frontal smile esthetics: A systematic review. American Journal of Orthodontics and Dentofacial Orthopedics 150: 740–750. 27871700 10.1016/j.ajodo.2016.06.022

[bibr26-14653125211034878] Pinho S Ciriaco C Faber J Lenza MA (2007) Impact of dental asymmetries on the perception of smile esthetics. American Journal of Orthodontics and Dentofacial Orthopedics 132: 748–753. 18068592 10.1016/j.ajodo.2006.01.039

[bibr27-14653125211034878] Raja HZ Nadeem A Awan HN (2016) Assessment of knowledge of anterior dental aesthetics amongst dental practitioners. Pakistan Oral & Dental Journal 36: 341–344.

[bibr28-14653125211034878] Rufenacht CR Berger RP (1990) Fundamentals of Esthetics. Chicago, IL: Quintessence Publishing Company.

[bibr29-14653125211034878] Sandler C Barry S Littlewood S Al-Musfir T Nazzal H (2019) Orthodontic management of traumatized teeth: A national survey of UK orthodontists. Dental Traumatology 35: 241–250. 31038825 10.1111/edt.12476

[bibr30-14653125211034878] Sarver DM (2004) Principles of cosmetic dentistry in orthodontics: Part 1. Shape and proportionality of anterior teeth. American Journal of Orthodontics and Dentofacial Orthopedics 126: 749–753. 15592225 10.1016/j.ajodo.2004.07.034

[bibr31-14653125211034878] Sharma AA Park JH (2010) Esthetic considerations in interdental papilla: remediation and regeneration. Journal of Esthetic and Restorative Dentistry 22: 18–28. 20136942 10.1111/j.1708-8240.2009.00307.x

[bibr32-14653125211034878] Shaw WC (1981) Factors influencing the desire for orthodontic treatment. European Journal of Orthodontics 3: 151–162. 6943029 10.1093/ejo/3.3.151

[bibr33-14653125211034878] Stone DH (1993) Design a questionnaire. BMJ 307: 1264–1266. 8281062 10.1136/bmj.307.6914.1264 PMC1679392

[bibr34-14653125211034878] Tsang S Royse CF Terkawi AS (2017) Guidelines for developing, translating, and validating a questionnaire in perioperative and pain medicine. Saudi Journal of Anaesthesia 11: S80–S89. 28616007 10.4103/sja.SJA_203_17 PMC5463570

[bibr35-14653125211034878] Tulloch JF Shaw WC Underhill C Smith A Jones G Jones M (1984) A comparison of attitudes toward orthodontic treatment in British and American communities. American Journal of Orthodontics 85: 253–259. 6584034 10.1016/0002-9416(84)90064-2

[bibr36-14653125211034878] Weiner SP Dalessio AT (2006) Oversurveying: Causes, consequences, and cures. In: Kraut AI (ed.) Getting action from organizational surveys: New concepts, methods, and applications. San Francisco, CA: Jossey-Bass, pp.294–311.

