# Supplementary material for: Orthodontic clinicians’ attitudes and knowledge of dentogingival aesthetics: A cross-sectional survey of BOS members
Source: J Orthod. 2021 Aug 11;50(4):400–9. doi: 10.1177/14653125211034878 (PMC10693736; doi:10.1177/14653125211034878)
Supplement: sj-docx-1-joo-10.1177_14653125211034878 – Supplemental material for Orthodontic clinicians’ attitudes and knowledge of dentogingival aesthetics: A cross-sectional survey of BOS members [file sj-docx-1-joo-10.1177_14653125211034878.docx]

**Supplemental Table 1** Checklist for Reporting Results of Internet E-Surveys (CHERRIES).

| **Item Category** | **Checklist item** | **Details** |
| --- | --- | --- |
| **Design** | Describe survey design | - Target population: Orthodontists in the UK - Sample frame: BOS members |
| **Institutional Review Board approval and informed consent process** | Approval  Informed consent  Data protection | - Ethical approval from King’s College London; College Research Ethics Committee. - Participants informed of estimated duration to complete the survey, anonymisation, investigators of the survey, aims of the study and ensured that participation is voluntary - Survey administered by BOS; no personal information collected. - Data collected by Online Surveys; certified to ISO 27001– the recognised information security standard. - All survey data protected with a password by research investigator. |
| **Development and pre-testing** | Development and testing | 1. Setting a clear research question and determining the research objective via an extensive literature search. 2. Item generation: all ideas and concepts considered for inclusion 🡪 item reduction to keep with aims of the study🡪 Questionnaire limited to six fundamental DG features through expert opinion 3. Questionnaire formatting:  - Clear instructions and headings to make questionnaire easy to follow - Related items grouped together - Multiple choice questions; answer available for selection - Ordinal responses to elicit respondent’s agreement with the statement - Free text: allows respondents to provide feedback  1. Testing and validation  - Content validity assessed by a panel of experts - Usability and technical functionality of the electronic questionnaire was tested prior to distribution of the questionnaire by piloting the survey |
| **Recruitment process and description of the sample having access to the questionnaire** | Open survey versus closed survey  Contact mode  Advertising the survey | - Open survey (survey was not protected with a password) - Email - None |
| **Survey administration** | Web/E-mail  Context  Mandatory/voluntary  Incentives  Time/Date  Randomization of items  Adaptive questioning  Number of Items  Number of pages  Completeness check  Review step | - Web based survey link sent to BOS members via email sent by BOS admin - The BOS is accepted as the sole national representative of all orthodontists - Voluntary - None - July-October 2020 - Items randomised - Not applicable - 12 items including one related to demographics - One - Completion of all knowledge and attitude-based questions were mandatory before the questionnaire could be submitted - Participants were able to scroll back to the previous questions to review or change their answers prior to submission |
| **Response rates** | Unique site visitor/ View rate/ Participation rate/ Completion rate | - This was calculated by the number of sent emails divided by the number of responses. |
| **Preventing multiple entries from the same individual** | Cookies used/ IP check/ Log file analysis /Registration | - Not used |
| **Analysis** | Handling of incomplete questionnaires  Questionnaires submitted with an atypical timestamp  Statistical correction | - Only completed questionnaires could be submitted and thus all the data analysed was based on completed questionnaires. - Time to complete the questionnaire was not measured. - Not used |

**Supplemental Table 2**

Orthodontic clinicians’ attitudes and knowledge of dentogingival aesthetics: an online survey

**What is your gender:**

Male

Female

**How old are you?**

<30

30-40

41-50

51-60

>60

**What year and country did you obtain your primary dental qualification in?**

**Year**

**Country**

-UK and Ireland

-European Union

-Other

**What year and country did you obtain your orthodontic speciality training in?**

**Year**

**Country**

-UK and Ireland

-European Union

-Other

**What level is your postgraduate qualification in orthodontics?**

Certificate

Diploma

Masters (taught)

Masters (research)

Doctorate (taught)

Doctorate (research)

**Where do you currently work? (please select all that apply)**

General dental practice

Specialist dental practice (Primary care)

Hospital employment (Secondary care)

Community dental service

Orthodontic registrar

Post-CCST Trainee

PhD student

NHS Consultant

University employee (e.g. Lecturer/ Senior Lecturer/ Reader/ Professor)

Armed forces

Other, please specify *______________*

**Do you have any special interests in dental aesthetics?**

Yes

No

**Have you attended any courses, lectures or seminars on dental aesthetics in the past 5 years?**

Yes

No

I don’t recall

**To the best of your knowledge can you select the answer that best fits the questions below:**

1. **What is the ideal gingival zenith position relative to the vertical bisected midline for each of the following teeth:**

**Maxillary central incisor:** *mesial coincident distal I don’t know*

**Maxillary lateral incisor:** *mesial coincident distal I don’t know*

**Maxillary canine:** *mesial coincident distal I don’t know*

1. **Which of the below statements is true for the ideal gingival margin level of the maxillary incisor teeth:**

*The gingival margin of the central, lateral and canine should all be at the same level*

*The gingival margin of the central and lateral incisors should be at the same level and the gingival margin of the canine should be at a lower level*

*The gingival margin of the central and canine should be at the same level and the gingival margin of the lateral incisor should be at a lower level*

*The gingival margin of the central incisor should be higher than the gingival margin of the lateral incisor and canine*

*I don’t know*

1. **An open gingival embrasure space (black triangle) is noticeably less aesthetic if it is more than:**

- *1mm*
- *2mm*
- *3mm*
- *4mm +*
- *I don’t know*

1. **What is the ideal connector area ratio between the maxillary central incisor, lateral incisor and canine?**

- *50:40:30*
- *40:30:20*
- *30:40:50*
- *50:50:50*
- *I don’t know*

1. **Ideally the incisal edge embrasure space between the maxillary central incisors, the central incisor and lateral incisors and the lateral incisors and canines should:**

- *Increase progressively distally from the midline*
- *Decrease progressively distally from the midline*
- *Decrease progressively mesially from the midline*
- *Be equal in size*
- *I don’t know*

1. **The dental midline can be deviated up to ______ before it is noticed by laypeople**

- *1 mm*
- *2 mm*
- *3 mm*
- *4 mm*
- *5 mm +*
- *I don’t know*

1. **When assessing orthodontic treatment outcomes, in your opinion how important is it to consider the following features:**

**Occlusion**

- Very important
- Fairly important
- Slightly important
- Not important

**Smile aesthetics**

- Very important
- Fairly important
- Slightly important
- Not important

**Dentogingival aesthetics**

- Very important
- Fairly important
- Slightly important
- Not important

1. **In your opinion, how much can orthodontic treatment influence dentogingival aesthetics?**

- Greatly
- Moderately
- Slightly
- No effect

1. **In your opinion how important are the above mentioned dentogingival features on overall smile aesthetics?**

- Very important
- Fairly important
- Slightly important
- Not important

1. **Which features in your opinion can have the greatest aesthetic impact on dental aesthetics?** (please select all the options that apply)

- Gingival zenith
- Gingival margins
- Gingival embrasure
- Connector area
- Incisal embrasure
- Incisal exposure at rest
- Gingival exposure on smiling
- Buccal corridors
- The number of maxillary teeth displayed on smiling
- Smile arc
- Dental midline
- Axial incisor angulation
- Midline diastema
- Crown width
- Crown height
- Golden proportions
- All the above
- None of the above

1. **Do you have any other comments that you would like to share with us?**
